# Transportation of dislocation plasticity in a dual-phase TiMo alloy

**DOI:** 10.1038/s41598-023-29057-2

**Published:** 2023-02-17

**Authors:** Jinghui Men, Xiaoqian Fu, Qian Yu

**Affiliations:** Center of Electron Microscopy and State Key Laboratory of Silicon Materials, Department of Materials Science and Engineering, Zhejiang University, 38 Zheda Road, Xihu District, Hangzhou, 310027 China

**Keywords:** Materials science, Structural materials

## Abstract

Understanding the coordinated deformation of multiple phases under applied stress is crucial for the structural design of dual-phase or multiphase advanced alloys. In this study, in-situ transmission electron microscope tensile tests were performed to investigate the dislocation behaviors and the transportation of dislocation plasticity during the deformation of a dual-phase Ti-10(wt.%) Mo alloy having hexagonal close-packed α phase and body-centered cubic β phase. We demonstrated that the dislocation plasticity preferred to transmit from alpha to alpha phase along the longitudinal axis of each plate, regardless of where dislocations were formed. The intersections of different α plates provided local stress concentration that facilitated the initiation of dislocation activities from there. Dislocations then migrated along the longitudinal axis of α plates and carried dislocation plasticity from one plate to another through these intersections as well. Since the α plates distributed in various orientations, dislocation slips occurred in multiple directions, which is beneficial for uniform plastic deformation of the material. Our micropillar mechanical testing further quantitatively demonstrated that the distribution of α plates and the α–α plates’ intersections plays important role in tuning the mechanical properties of the material.

## Introduction

To improve the materials’ mechanical properties, dual-phase and multiphase structures are frequently developed and manufactured^1^. The combination of different phases would be beneficial to material’s overall performance because each phase has distinct structures and properties^2,3^. As a result, alloys made up of two or more phases have been widely used in essential industries including aerospace and industrial engineering^4,5^.

Two essential mechanical characteristics that have a direct impact on how well multiphase materials function in service are strength and plasticity^6,7^. Notably, the way plastic strain is transported across various phases when stress is applied is intimately related to strength and plasticity^8,9^. Since different phases have varied deformability, it is always quite interesting to understand how several phases accommodate plastic strain together for materials including two or more phases. There had been numerous attempts to end this relationship. Edalati et al. examined the behavior of face-centered cubic (FCC) and body-centered cubic (BCC) structures under high-pressure torsion using dual-phase AlFeCoNiCu alloy. During plastic deformation, twins and stacking faults emerged in the FCC, while dislocation slips happened in the BCC^10^. According to research by Tu et al., ferrite was crucial to the deformation of bainite-polygonal ferrite dual-phase pipeline steel. The slip systems of {123} 〈111〉 and {112} 〈111〉 were initially turned on in ferrite. Later, strain concentration caused the gliding of a new slip system {110} 〈111〉 to be seen in both the bainite and ferrite phases, which was supposed to be proof of strain transit from ferrite to bainite^11^. Commercial titanium alloys are mostly made up of α phase with hexagonal close-packed (HCP) structure and β phase with BCC structure. Typically, both phases are formed into plates. Generally, the α phase deforms before the β phase since the α phase is thought to be softer. The deformation of the β phase occurs later, which should coordinate with the deformed α plates to accommodate strain^12,13^. However, the actual process of transporting plastic deformation within this dual-phase structure has not yet been revealed.

In the present paper, in-situ transmission electron microscope (TEM) straining testing was performed to directly observe the dislocation glide within the dual-phase structure of titanium alloy. It was found that dislocations moved along the plate’s longitudinal directions for the α phase. The intersection of different α plates generated certain points with local stress concentration that facilitated the transfer of dislocation activities from one plate to another. Most of the transferred dislocations moved along the longitudinal direction, and some slipped in multiple directions. Due to the variable orientations of α plates, the generation of dislocation slips can be in multiple directions, which would benefit homogenous plastic deformation.

## Results and discussion

### Microstructure of dual-phase TiMo alloy

**Figure 1:**
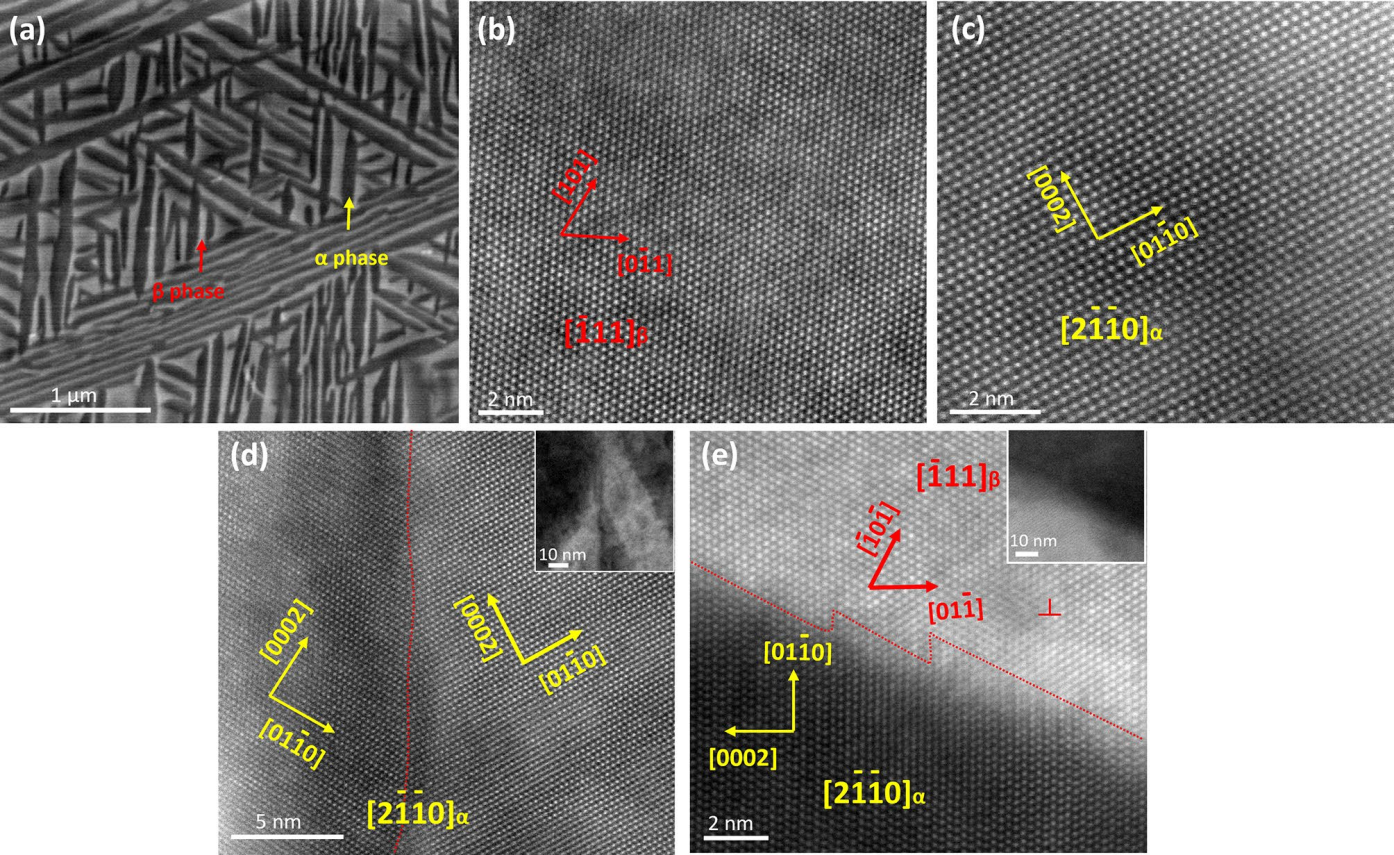
Microstructure of the dual-phase TiMo alloy. (**a**) Scanning electron microscopy image showing the homogeneous distribution of α and β phases. (**b**,**c**) HAADF images showing the atomic structure of β phase and α phase, viewed along $[\overline{1 }11]$_β_ and $[2\overline{1 }\overline{1 }0]$_α_, respectively. (**d**) Atomic resolution HAADF image showing the α–α interface viewed along $[2\overline{1 }\overline{1 }0]$ orientation. (**e**) Atomic resolution HAADF image showing the α–β interface viewed along $[2\overline{1 }\overline{1 }0]$_α_//$[\overline{1 }11]$_β_ orientation.

The typical microstructure and atomic structure of the dual-phase TiMo alloy are presented in Fig. 1a–c. The two phases had distinct contrasts and different shapes. The atomic structures of the two phases shown in the high-angle annular dark-field (HAADF) image illustrated that the dark plates were associated with the HCP structure and the bright plates with the BCC structure, corresponding to the α phase and β phase, respectively. The clear atomic arrangement was acquired from zone axis $[\overline{1 }11]$ of β and $[2\overline{1 }\overline{1 }0]$ of α at a 2 nm scale. Furthermore, the two phases were distributed homogeneously and interlaced. Specifically, the α phase was in the form of a submicron-sized plate and partitioned the bright β phase into comparative-size plates and blocks. Notably, the plate-shaped phase exhibited a short side with a width of about 80 nm and a longitudinal direction with a length of up to several micrometers. As such, α–β interfaces and α–α intersections were considered the main barriers to the transportation of dislocation plasticity in the dual-phase TiMo alloys. The atomic structures of α–α intersections and α–β interfaces were characterized and displayed in Fig. 1d,e, respectively. It was shown that two α grains at the α–α interface were symmetric and well fitted without any stress concentration. Meanwhile, the α–β phase boundary demonstrated coherent character with a classical Burgers orientation relationship of 〈$11\overline{2 }0$〉_α_//〈$1\overline{1 }1$〉_β_ (Fig. 1e)^14^. A dislocation with a Burgers vector of 1/2〈110〉 (marked by “⊥”) appeared close to the boundary instead of lying at the boundary. It was noted that the coherent interface between nanoparticles and matrix with low mismatch and little lattice distortion could effectively facilitate the release of stress concentrations, generating considerable plastic strain^15^. The high coherency of α–β interfaces and α–α intersections implied limited resistance for dislocation transferring.

### Strain transportation in dual-phase architecture

**Figure 2:**
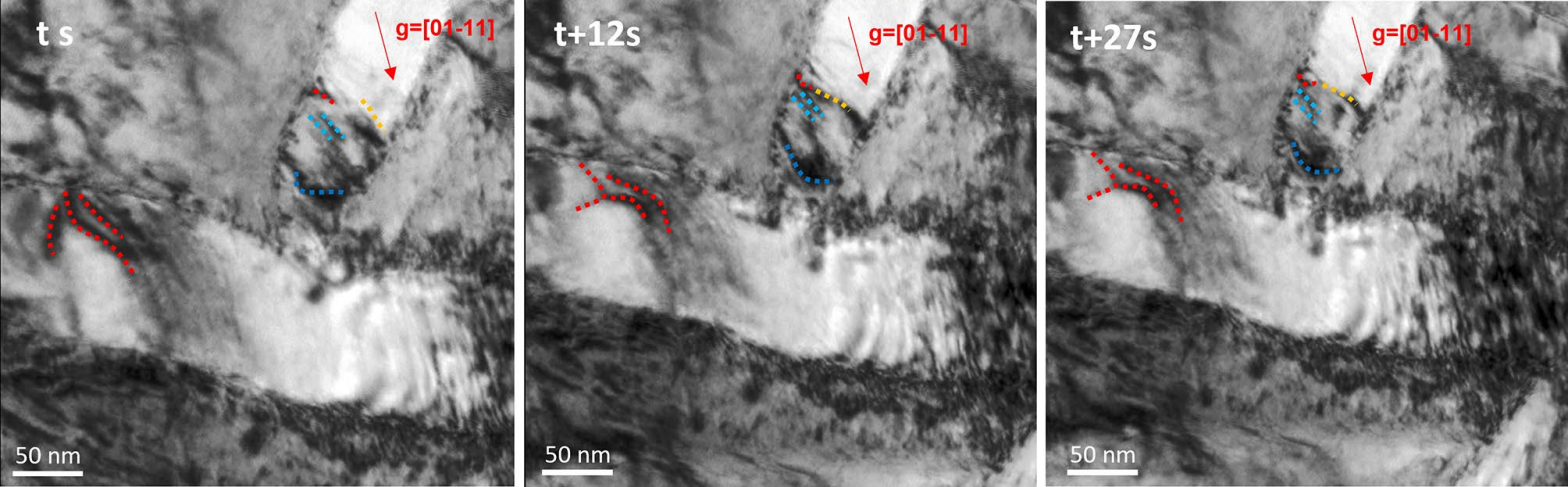
Transportation of dislocation activities between different α plates. Serial TEM images showing dislocation glide along the longitudinal direction of each α phase.

We performed in situ TEM straining tests to observe the dynamic dislocation behaviors within the two phases and directly revealed the transportation of dislocation plasticity within this dual-phase structure. According to the observation over a wide range of strains, it was determined that dislocations were preferentially activated in the α phase during the early stage of deformation, as shown in the serial images captured from Supplementary Movie 1 in Fig. 2. These pictures were acquired at the [$11\overline{2 }0$] zone axis with the [$01\overline{1 }1$] g vector. The fast movement of dislocations in α plates dominated the deformation process at the initial stage, which was attributed to the densely packed atoms in the α phase^16^ and the lower critical shear stress for dislocation slip in the HCP structure^13^. Interestingly, the dislocations activated in the α phase tended to slip along the longitudinal direction of α plates in most cases. As shown in Fig. 2, dislocations glided along the longitudinal directions in both α plates, although the two distinct plates oriented orthogonally. In the horizontal plate, dislocations (marked with red dashed lines) moved from left to right. Moreover, the dislocations (marked by dashed lines with other colors) moved from top to bottom in the vertical plate. Overall, the dislocations in the α phase glided along the longitudinal direction of the plates. As α plates oriented in multiple directions and intersected with other α plates, dislocations activities occurred in multiple directions, resulting in homogeneous deformation comparative to only one or limited preferential slips. Generally, the Schmid factor determines which grain activates the dislocation first and which slip system is activated^17^. In this case, the dislocations preferentially glided along each plate’s longitudinal direction, especially in the α phase. It was also noted that the width and length of each alpha plate reside at about 80 nm and several micrometers, respectively. The shape characteristics could result in anisotropic properties in the two directions, including Young’s Modulus, yield strain, and tensile strength^18^. Therefore, it was expected that the anisotropic shape could result in an anisotropic Hall–Petch effect, affecting the movement of dislocations in the α phase. And the result was consistent with our observation.

**Figure 3:**
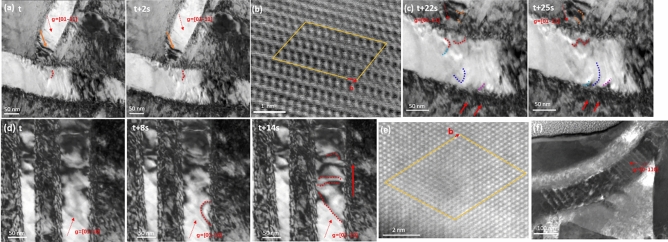
Dislocations glide along longitudinal direction. (**a**) Dislocations were excited from α–α junction and moved along the longitudinal direction of α plates. (**b**) The characteristic of dislocation in α phases along longitudinal direction. (**c**) Dislocations activities were excited from α–α junction and moved in multiple directions. (**d**) Dislocations activities at the α–β boundary and their movement along α phase. (**e**) The characteristic of dislocation near α–β phase boundary (**f**) Post-mortem STEM image showing the glide of dislocations along the longitudinal direction of α plates in bulk counterpart as well.

By making close observation of the origin of dislocations in the α phase, it was found that the dislocation activities were generated from both α–α junctions and α–β interfaces, and they glided along the longitudinal direction of α plates. For the α–α junction, most dislocations glided along the longitudinal direction of the α strip (Fig. 3a and Supplementary Movie 2). The dislocations first piled up at the junction (marked by an orange arrow in Fig. 3a), causing stress concentrations. The Burgers vector of the dislocations in the α phase was 1/3〈$11\overline{2 }0$〉, and the slip plane was {0001} as characterized by Burgers circuit analysis, as shown in Fig. 3b. It was also noted that a few dislocations activated from α–α junctions glided in multiple directions (Fig. 3c). These dislocations moved toward the α–β phase boundary and were impeded by the phase boundary. As the dislocations interacted with the α–β phase boundary, dislocation activities were initiated in the neighboring β phase (pointed out by red arrows). In this case, the activation of dislocations in the relatively hard β phase might result from the coherency of the α–β phase boundary, which could provide channels for dislocations to slip through. Consequently, both phases were plastically deformed and contributed to homogeneous deformation. For the α–β phase boundary, as the applied stress increased, it was observed that dense dislocations were located at the α–β phase boundary and could be de-pinned under the applied stress, as marked by the red dashed line in Fig. 3d. For instance, from t + 8 s to t + 14 s, the high density of the newly excited dislocations generated from the α–β phase boundary moved along α plates (indicated by the red arrow). These pictures were acquired at the [$11\overline{2 }0$] zone axis with the [$01\overline{1 }0$] g vector. According to the atomic resolution HADDF image, it could be found the Burgers vector of the dislocations near the α–β phase boundary was 1/2〈$\overline{1 }11$〉, and the slip plane was {$110$} as characterized by Burgers circuit analysis, as shown in Fig. 3e, corresponding to edge dislocation. Post-mortem characterizations of the dislocation structure of the deformed sample (shown in Fig. 3f) also illustrated that the dislocations regularly lined up along the α stripe at low strain. The picture was acquired at the [0001] zone axis with [$0\overline{1 }10$] g vector under the TEM model. The dislocation plasticity primarily transports in the α phase along the longitudinal direction, regardless of where the dislocation originated.

### Micropillar compression test

**Figure 4:**
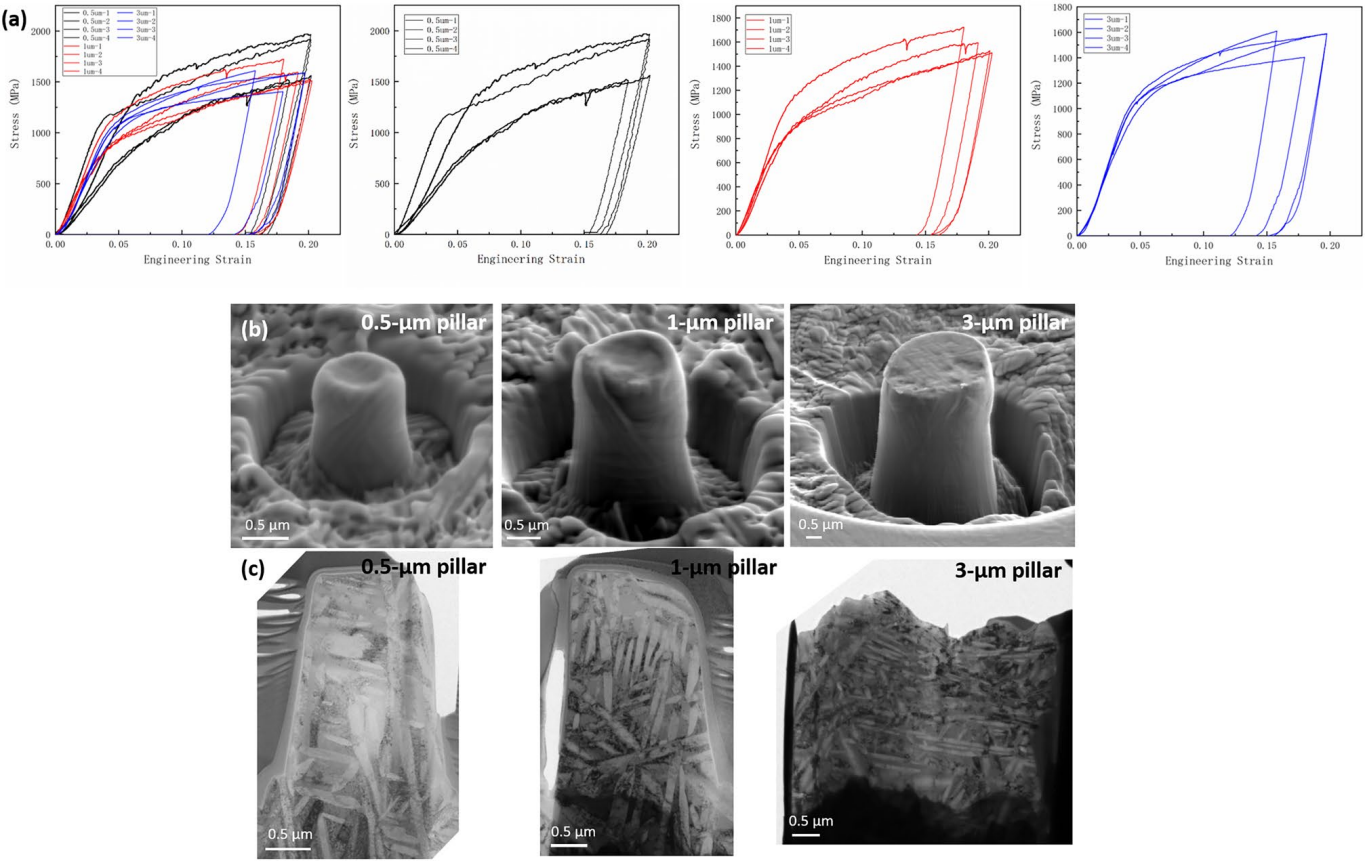
Mechanical properties of TiMo micro-pillars with different sizes. (**a**) Stress–strain curves of micro-pillars with diameter of 0.5 μm, 1 μm, and 3 μm. (**b**) SEM images for three sized micro-pillars after compression, all pillars deformed uniformly. (**c**) Cross-section TEM images for the micro-pillars with different sizes after compression.

It has been well-accepted that strain transportation within the dual-phase structure is critical in accommodating deformation and strengthening. It was considered that the dislocation activities transmitted through the α–β phase boundary in the dual-phase alloy since it was the key interface with the largest area. While in this case, it was found that α–α junctions played an important role in transporting dislocation plasticity at the early stage of plastic deformation. The longitudinal direction of the α phase could be regarded as the main channel for dislocation slip, and the transportation of deformation was accomplished by the dislocation transfer between various α plates through the α–α junctions. Therefore, it was speculated that the validity of α–α junctions should be a significant factor affecting the mechanical properties of materials. To quantitatively analyze the contribution of such structures to the mechanical properties, micropillars with a diameter of 0.5, 1, and 3 μm were prepared and compressed (see Methods). Four engineering stress–strain curves for each sized micropillar from the in-situ SEM compression tests were plotted in Fig. 4a. The steady serrated curves signified that all dual-phase TiMo pillars were deformed continuously without strain burst, indicating superior plastic stability. In addition, all pillars displayed uniform deformation since all pillars deformed into a bulged shape after compression (Fig. 4b). The high deformation stability of the dual-phase pillars resulted from the architecture of the two phases. Interestingly, the different-sized pillars displayed yield strengths in a similar range. However, data was more scattered at smaller sizes, distinct from the traditional size effect of single-phase metals, i.e., demonstrating that the reduction of sizes dramatically enhances strength^19,20^. The 3 μm pillars exhibited about equal yield strength at 1050 MPa, and the 1 μm pillars displayed variations in yield strength from 1000 to 1200 MPa, while the yield strength of 0.5 μm pillars fluctuated from 900 to 1300 MPa. Note that the dislocations were first activated in the α phase during the plastic deformation of the dual-phase alloy. The size of the α plates, especially the length in the longitudinal direction, determined the yield strength. Using FIB to extract cross-sections from the deformed pillars, it was observed that the length in the longitudinal direction of α plates and the number of α–α intersections contained in the micropillars varied specifically at the smallest tested size (Fig. 4c). In large pillars, the number of α plates was significant. Therefore, the mechanical data was closer to the statistical behavior. However, as the sample size decreased, although the yielding of the pillar was still related to the dislocation activities within α plates, the mechanical data varied since the distinct shape and orientation of each α plate and how different α plates intersected had a great influence on the transportation of dislocation plasticity. Thus, the yield strength in the smaller pillar showed considerable fluctuation.

## Conclusions

We gained insight into the transportation process of dislocation plasticity within the dual-phase structure using in-situ TEM straining testing and compression experiments on a dual-phase TiMo alloy.

1. The dislocation glide was preferentially initiated in the α phase along the longitudinal direction of α plates. This is considerably related to the shape-induced Hall–Petch effect.
2. The dislocation plasticity was transported from α to α plates through the α–α intersections at the early stage of plastic deformation and was transported from the α to β phase later through the dislocation interactions at the phase boundaries. It should be noted that the dislocations can either be generated from the local stress concentrations along the phase boundaries and the phase intersections or directly transmitted from the adjacent phases. Identifying the two cases remained a great challenge even though the Burgers vectors of the in and out dislocations were known.
3. In such a way, dislocations glide could be activated in multiple directions, benefiting homogenous plastic deformation. Thus, the distribution of different phases became an essential factor that can tune the plastic deformation of materials. Also, homogenous plastic deformation may be achieved if the distribution of phases was well designed and produced

The results provide fundamental insight for understanding and designing dual-phase materials with optimized mechanical properties. We believe this fundamental finding could benefit alloy design and manufacture. By tuning the Hall–Petch effect, phase distribution, and boundary characters, special mechanical properties can be achieved through microstructure engineering.

## Methods

The experimental material was Ti-10Mo (wt. %) alloy, which was first solution treated at 1000 °C for 1 h and aged at 600 °C for 10 min to obtain a dual-phase structure with coexistence of α and β phases, then followed by quenching to room temperature. The thin TEM specimens were fabricated by using a FEI Quata 3D FEG type dual-beam focused ion beam (FIB) instrument. The atomic structure of the TiMo alloy specimen was characterized by spherical aberration-corrected scanning transmission electron microscopy (FEI Titan G2 80–200 ChemiSTEM) with an accelerating voltage of 200 kV. The TEM samples for the in-situ tensile test were thinned by PIPS instrument in order to obtain electron-transparent area. The specimen was adhered to a substrate at ambient temperature and placed for 24 h. Lastly, the specimen was strained by using a Gatan 671 low-temperature tensile holder at ambient temperature in a FEI Tecnai G2 F20 S-TWIN TEM with an accelerating voltage of 200 kV. The micro-pillars for the compression tests were prepared using the above FIB instrument and the pillars were processed into three sizes (0.5 μm, 1 μm and 3 μm, respectively). The in-situ compression experiments were performed in FIB with a Hysitron PI 87 micro-indenter in a displacement-control mode and the displacement rate was 5 nm/s.

## Supplementary Information

Supplementary Legends.

Supplementary Movie 1.

Supplementary Movie 2.

## Data Availability

The datasets used and/or analysed during the current study available from the corresponding author on reasonable request.
