# Supplementary material for: Transportation of dislocation plasticity in a dual-phase TiMo alloy
Source: Sci Rep. 2023 Feb 17;13:2829. doi: 10.1038/s41598-023-29057-2 (PMC9938182; doi:10.1038/s41598-023-29057-2)
Supplement: Supplementary file 1 — Supplementary Legends. [file 41598_2023_29057_MOESM1_ESM.docx]

**Supplementary Materials**

**Transportation of dislocation plasticity in a dual-phase TiMo alloy**

**Jinghui Men^1^, Xiaoqian Fu^1^, Qian Yu***

*^1^* *Center of Electron Microscopy and State Key Laboratory of Silicon Materials, Department of Materials Science and Engineering, Zhejiang University, Hangzhou 310027, China.*

***Correspondence to:** Prof. Qian Yu, Center of Electron Microscopy and State Key Laboratory of Silicon Materials, Department of Materials Science and Engineering, Zhejiang University, 38 Zheda Road, Xihu district, Hangzhou 310027, China. E-mail: [yu_qian@zju.edu.cn](mailto:yu_qian@zju.edu.cn)

**Supplementary Movie 1.** In situ tensile test of dual-phase TiMo alloy showing the movement of dislocations along the longitudinal direction of α plates.

**Supplementary Movie 2.** In situ tensile test of dual-phase TiMo alloy showing the actived dislocations origin from α-α junction and move along the longitudinal direction of α plates.
